# Editorialets

**Published:** 1908-01

**Authors:** 


### Editorialets.

Dr. J. F. Bailey, of Waco (Osteopath), has been appointed on the State Board of Medical Examiners, vice Collins, resigned. This is an excellent appointment; the best the Governor could have made, in the opinion of the "Red Back."

Dr. J. M. Inge, of Denton, was elected President of the North Texas Medical Society at its recent meeting at Fort Worth. That great society held a rousing and enthusiastic meeting, and they had a swell banquet. Our esteemed contemporary (*Texas Courier-Record*) has a fine writeup of the meeting.

Among the doctors from Texas who are doing post-graduate work at the New Orleans Polyclinic are Dr. M. Rives, Houston; Dr. J. D. Phillips, Tyler; Dr. J. L. Guest, Lockney; Dr. J. R. Kight, San Angelo; Dr. J. W. Miller, Hillsboro; Dr. A. M. Anderson, Kennedy, and Dr. M. B. Canon, Moscow.

DR. H. N. GRAVES, the popular and well known "serum" apostle, for many years detail man for Stearns & Co. (who "drapped their money pu's" when they let him go), is now traveling in Texas in the interest of Parke, Davis & Company. It is a safe bet that the "sweet singer" has more friends to the square mile than any man in Texas. His zeal in the cause of serum therapy is only excelled by his profound knowledge of the subject and its literature. Parke, Davis & Company are indeed fortunate in securing his services.

---

A BOARD OF COMMISSIONED MEDICAL OFFICERS will be convened to meet at the Bureau of Public Health and Marine Hospital Service, 3 B Street S. E., Washington, D. C., Monday, January 20, 1908, at 10 o'clock a. m., for the purpose of examining candidates for admission to the grade of assistant surgeon in the Public Health and Marine Hospital Service.

Candidates must be between 22 and 30 years of age, graduates of a reputable medical college, and must furnish testimonials from responsible persons as to their professional and moral character. Write for details if interested.

---

AMONG MY NUMEROUS CHRISTMAS GIFTS, there was none more acceptable than a shipment of Listerine and Dermatic Soap with the compliments of the manufacturers, Lambert Pharmacal Co. Listerine is simply indispensable in the family. It is the best intestinal antiseptic I know and almost a specific in nausea, diarrhea, etc.; it is the best antipruritic, and after shaving, or bath, it is a delightful and soothing application. As a gargle, or mouth wash, it is cleansing, healing and delightful. No family should be without it.

We acknowledge, too, with thanks, an acceptable little present of a leather pocketbook from Antiphlogistine—the Denver Pharmacal Co., together with a paid-up accident policy for $2500.

---

JOURNALS MAY COME and journals may go, but the "Red Back" goes on forever (with apologies to Tennyson). Notwithstanding the stringency, and the efforts of the Octopus and ye council to boycott us, the old guard is standing pat and swearing by it, while new fellows are subscribing freely. And my ad. patrons are standing by me like little men. One of the biggest and best New York agencies (O'Gorman), in renewing contract for 1908 for three pages, and paying *for it in advance,* writes: "While numerous changes and eliminations have taken place in the lists concerned, you will note that the 'Red Back' is not affected thereby."

Black Eye for National Department of Public Health.—Mr. Roosevelt has written to Dr. Fisher, Chairman of the A. M. A. Committee of One Hundred, whose mission was to secure legislation in the interest of public health, specifically, an act of Congress creating a Department of Public Health with a Secretary, in the President's Cabinet, expressing himself as opposed to such measure, and says that the country does not need a Secretary of Public Health; that there are Cabinet officers enough. Mr. Roosevelt says that a bureau (a kind of sub) in one of the departments is sufficient. Well, we have that, and what does it amount to? Really, it looks as if it were not "worth while" to try to open the eyes of our lawmakers to the fact that the public health is paramount, and that all else depends upon it. Heigh ho!

In Memoriam.—Frederic Roberts, M. D., Medical Department University of Tennessee, 1907. Born in Nashville, Tennessee, October 13, 1886; youngest son of Dr. Deering J. and Mrs. Rachel Lavina Roberts. Died at the family residence on Linden Avenue, Nashville, at 11 a. m., Sunday, December 8, 1907.

The Journal extends sincerest sympathy to its old friend Roberts, the Nestor of the medical press.

"We will be patient, and assuage the feeling
We may not wholly stay,
By silence sanctifying,—not concealing
The grief that must have sway."—D.

Christmas Remembrances.—The heart of ye editor was made to rejoice Christmas in many ways. Renewal and "continue till forbid" contracts, many and prompt responses to his suggestion that the north wind doth blow and we shall have snow and that coal is $10 a ton; also, that Betty and the baby needed shoes and stockings; by numerous presents; by numerous and warm and cordial letters of appreciation of the efforts of the "Red Back" to sustain and defend the honor of legitimate medicine and to instigate to unity, peace and concord in our own ranks; to eschew politics; to stand by our State Association, and to labor still in the cause of humanity; and the best wishes of hosts of attached friends for the continued success and prosperity of ye "Red Back"

and its editor and his *better nine-tenths.* God bless them all. They have, indeed, been loyal, and the deserters, instigated by you know whom, are as rare as hen's teeth.

---

A PROPOSED SUBSTITUTE FOR CASTRATION.—Elsewhere in this issue, under the head of "Procreation of Defectives," is a letter to the *Boston Medical and Surgical Journal,* in which it is claimed that complete sterility in the male can be produced by the X-ray, and the writer writes me a personal letter to the effect that he witnessed a demonstration of the fact upon a healthy normal man under the hands of Dr. F. B. Granger, 591 Boylston, Beacon Street, Boston, and Dr. W. F. Whitney of the Harvard Medical School.

Read the article. The writer says: "The treatment is painless, does not produce impotence nor decrease the pleasure derived from sexual intercourse." This fact, of course, puts the treatment out of the question as applicable to the rapist; it may do for defectives, if the law should ever sanction any method of limiting the output of insane and criminals by heredity, but for the rapist a clean cut is the only treatment. We will get such a law in Texas next session of the Legislature.

---

A SPECIMEN LETTER.

STONEBURG, TEXAS, December 16, 1907.

*Dr. F. E. Daniel, Editor "Red Back," Austin, Texas.*

DEAR DOCTOR: Enclosed find $2.00, for which please advance my subscription two notches. In renewing for the tenth consecutive year, I desire to say to you that the "Red Back" is the most welcome visitor that comes to my library. Especially do I want to take "ye editor" by the right hand and heartily endorse the course he has pursued toward the "Octopus," which I consider the enemy, rather than the friend, of the individual doctor. Also I desire to commend the stand he has taken against the "monster evil," whisky, which is the foundation of a large majority of crimes among the American people today.

Long live the "Red Back." A Merry Christmas and a Happy and Prosperous New Year to "ye editor."

Respectfully,

L. P. TENNEY.

---

SOUTHWESTERN (TEXAS) INSANE ASYLUM, SAN ANTONIO.—The Governor has received the sixteenth annual report of Dr. W. L. Barker, Superintendent of the Southwestern Insane Asylum at

San Antonio, for the fiscal year ending August 31, 1907. The report goes into the details of the doings of that institution. In a preliminary report the board complains of the lack of sufficient room to accommodate the rapidly increasing population of the institution. It is stated that the institution can accommodate 700 patients, but 100 above that number has been crowded in. Recommendation is made that the asylum's capacity be increased to 1200 patients. In his report, Superintendent Barker says:

"Many insane are crowded in jails and are denied the benefit and care they would receive in the institution. After remaining in jail from six months to a year these cases are almost invariably hopeless, and then, when they are admitted to the institution, they are there to stay as chronic, incurable cases."

In addition the board recommends a separate building for the care of different sexes of infirmary patients. This class of patients of both sexes is cared for in the same ward. Tubercular patients are also cared for in the infirmary hospital and recommendation is made that a complete separation of tubercular patients be made from all others.

Dr. Barker in his report begins with financial matters, saying there was $139,980 appropriated by the Legislature for maintenance. Of this $124,362.91 was expended, leaving a balance of $15,617.09 in the hands of the Comptroller. There was also deposited in the treasury $5,450.96 cash collected from non-indigent patients, making a grand total of $21,068.05 to the credit of the institution.

The daily average attendance has been 747 and the annual per capita cost was 43 cents a day. This is a little higher than the cost of last year, as the cost of some of the provisions has advanced. One of the items mentioned is fuel oil, which has advanced 50 cents a barrel.

At the end of the fiscal year in September, 1906, there were 808 patients in the asylum. A total of 91 patients were admitted during the year; a total of 897 were treated during the year. During the year 51 patients died and 29 were discharged. There remained on August 31, 1907, 817 patients.

One improvement in the San Antonio asylum over other asylums is the dairy. During the year the dairy produced 58,938 gallons of milk and 12,250 pounds of butter, which more than met the demands of the institution. Hogs were fattened from the waste food at the asylum and during the year there were killed for the use of the institution 24,640 pounds of pork. The dairy facilities have been improved recently and during the last two months the dairy produced 50 pounds of butter a day. During the year it has not been necessary to purchase a chicken or an egg for the institution. A total of 385 chickens have been killed and 3101 dozen eggs used. The apiary produced 600 pounds of strained honey. A total value of $23,616.85 worth of supplies has been raised for the institution. A mattress house has been built and a mattress maker placed in charge, mattresses being made of moss.

